# Adhesion to Zirconia: A Systematic Review of Current Conditioning Methods and Bonding Materials

**DOI:** 10.3390/dj7030074

**Published:** 2019-08-01

**Authors:** Daniele Scaminaci Russo, Francesca Cinelli, Chiara Sarti, Luca Giachetti

**Affiliations:** Department of Experimental and Clinical Medicine—Unit of Dentistry, University of Florence, Via del Ponte di Mezzo, 48-50127 Firenze, Italy

**Keywords:** zirconia, composite resin, adhesion, bond strength, systematic review

## Abstract

*Background.* Reliable bonding between resin composite cements and high strength ceramics is difficult to achieve because of their chemical inertness and lack of silica content that makes etching impossible. The purpose of this review is to classify and analyze the existing methods and materials suggested to improve the adhesion of zirconia to dental substrate by using composite resins, in order to explore current trends in surface conditioning methods with predictable results. *Methods.* The current literature, examining the bond strength of zirconia ceramics, and including in vitro studies, clinical studies, and a systematic review, was analyzed. The research in the literature was carried out using PubMed and Cochrane Library databases, only papers in English, published online from 2013 to 2018. The following keywords and their combinations were used: Zirconia, 3Y-TZP, Adhesion, Adhesive cementation, Bonding, Resin, Composite resin, Composite material, Dentin, Enamel. *Results.* Research, in PubMed and Cochrane Library databases, provided 390 titles with abstracts. From these, a total of 93 publications were chosen for analysis. After a full text evaluation, seven articles were discarded. Therefore, the final sample was 86, including in vitro, clinical studies, and one systematic review. Various adhesive techniques with different testing methods were examined. *Conclusions.* Airborne-particle abrasion and tribo-chemical silica coating are the pre-treatment methods with more evidence in the literature. Increased adhesion could be expected after physico-chemical conditioning of zirconia. Surface contamination has a negative effect on adhesion. There is no evidence to support a universal adhesion protocol.

## 1. Introduction

In recent decades, the increasing aesthetic needs in dentistry have led to the progressive overcoming of metal-ceramic prosthesis and led to a focus on indirect metal-free restorations. Zirconia has occupied an increasingly important role, thanks to its excellent mechanical [1] and biocompatible characteristics [2]. Initially, this material was used only for all-ceramic frameworks. Subsequently, the introduction of translucent zirconia on the market, with improved aesthetic properties [3,4,5], allowed for the realization of monolithic prosthetic products with innumerable advantages: elimination of chipping risk, good mechanical properties (superior to monolithic lithium disilicate products), the possibility of processing by a CAD-CAM technique (greater standardization and quality of results, with cost reduction), manufacturing of smaller thicknesses, and a more conservative dental preparation [6]. Unfortunately, zirconia, unlike glass ceramics, is not susceptible to etching and this makes it impossible to realize the adhesive procedures. Realizing safe and standardized adhesive cementation protocols of zirconia is necessary in order to adequately complete the conservative/prosthetic treatment plan, especially when the preparation is not retentive, (due to the characteristics of the abutment or of the prosthesis design), or when it is necessary to improve the mechanical characteristics of the tooth-prosthesis complex.

Over the last few years, many adhesion techniques have been studied. Different treatments of the zirconia surface, application of primers or adhesives, and various types of resin cements have been tested. However, a standardized adhesive cementation protocol, that provides univocal and reliable results, has not been identified [7,8,9].

The data we have available today come mostly from laboratory studies that, although they are useful for guiding subsequent clinical trials, have limitations in terms of clinical evidence. Furthermore, the results obtained from such a large number of tested techniques are not directly comparable. It is difficult to generalize the results in relation to the zirconia sample, or to the materials used, considering the wide range of products available on the market.

**Figure 1:**
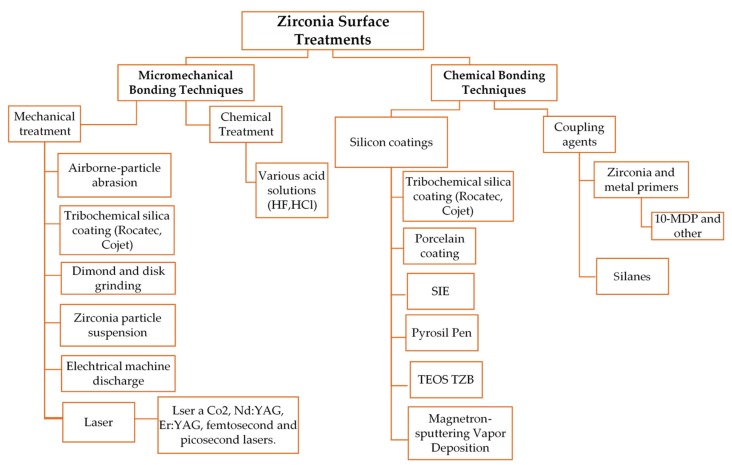
Zirconia surface treatments.

The aim of the review is, therefore, to compare different treatments of the zirconia surface, in order to determine a valid operative protocol for adhesive cementation. The main zirconia treatments are summarized in Figure 1.

## 2. Materials and Methods

### 2.1. Search Strategy

To review the literature, the National Library of Medicine database was consulted using PubMed. The research was carried out on 2 January 2019 and the studies published from 1 January 2013 to 31 December 2018 were selected. The Cochrane Library database was also analyzed with a limitation on publication year (2013–2018). It was decided to choose this time interval to get a picture of what is the current knowledge on the subject discussed, regarding the new materials recently put on the market. Studies regarding the evaluation of the bond between zirconia and composite resins have been included in the review. The following terms and their combination were searched: “Zirconia,” “3Y-TZP,” “Adhesion,” “Adhesive cementation,” “Bonding,” “Resin,” “Composite resin,” “Composite material,” “Dentin,” and “Enamel.” The research includes laboratory studies, clinical studies, and systematic reviews. 

### 2.2. Eligibility Criteria

Regarding laboratory studies, no exclusion criteria were set in relation to the type of test performed for the evaluation of the bonding strength. However, it is important to evaluate the ability of the adhesive bond to resist over time. In this regard, studies in which samples are subjected to at least 5000 thermocycles or at least one month of H_2_O storage are included in the review. Regarding clinical trials, RCTs and observational studies were included, with a follow-up, at least, of 5 years. The examined articles evaluate the clinical performance of adhesively cemented zirconia prostheses, in particular anterior cantilever prostheses, and prosthesis on inlays in the posterior sector. Studies that analyze traditional bridges with full crowns on the abutment teeth have been excluded. Inclusion criteria are listed in Table 1.

## 3. Results

The research carried out in PubMed (Table 2 and Table 3) and the Cochrane Library (Table 4), 370, 77, and 31 studies are obtained, respectively. The duplicates are eliminated, obtaining a total number of 390 studies. By reading the abstract, studies that are not considered relevant, those that do not meet the aging requirements, or do not meet the inclusion criteria are discarded. Regarding in vitro studies, the most common reasons for elimination were the absence of the evaluation of the bond strength and the lack of evaluation of the aging effect (no TC or TC <5000). Some studies have been eliminated because they are not relevant (e.g., adhesion of zirconia brackets or posts) or not pertinent because they do not evaluate zirconia-resin bond (e.g., bacterial adhesion to zirconia). Pilot studies and case reports have also been discarded. After this screening, 93 studies are subjected to a full-text examination.

**Figure 2:**
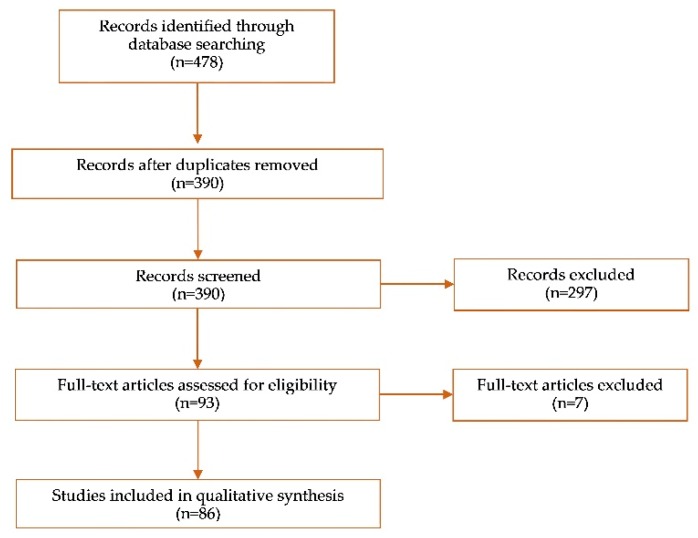
Studies selection process.

The articles found consist mainly in laboratory studies. The clinical studies are in small numbers. Eight systematic reviews were also selected, including seven that were subsequently discarded following their full reading, since they do not meet the exclusion criteria of this review. The selected studies are summarized in Table 5 and Table 6. Table 7 lists the eliminated reviews, specifying the reason for their exclusion. Figure 2 shows the study selection process.

## 4. Discussion

The studies examined in this review mainly consist of laboratory studies. Five clinical studies were also found, while most of the initially included systematic reviews were eliminated because their inclusion criteria do not reflect the limits set for this review. It was decided to review various types of articles, in order to have an overall view of the current knowledge regarding the adhesion of zirconia.

Different types of tests are performed to estimate the bond strength. The most widely used is the Macro Shear, which is the easiest to set up. However, it must be considered that the type of test can partly influence the result. The preparation of the Macro Test leads to a greater heterogeneity in the distribution of stress, due to the wider adhesive interface [98]. The µShear and µTensile show lower variation coefficients and offer the possibility of analyzing different regions of the same sample [98]. In terms of results, this leads to higher bond strength values, since the smaller the area, the lower the possibility of finding a defect that limits the bond [98]. Given the heterogeneity of the results, it was decided not to directly compare the bond strength values obtained in the studies.

Long-term water storage and thermocycling are commonly used methods of artificial aging that affect the resin bond to ceramic [67,73]. This review includes both methods because, although thermocycling seem to be more reliable, contradictory results have been reported [67]. While water storage simulates aging due to water uptake and hydrolytic degradation, thermocycling represents in vitro hydrothermal aging [7]. The number of cycles varied greatly in the in vitro studies [7], which is a standardized protocol for thermocycling that permits a comparison across different studies that is not available [67]. It was decided to match the ISO norm 10477, where the minimum number of TC was proposed 5000, to assess metal-resin bond [7]. The increased number of cycles above 5.000 that was up to 10.000 or 20.000 do not significantly affect the result [15]. The frequency of cycling in vivo remains to be determined at present and requires formal estimation [7,8,9,10,11,12,13,14,15,16,17,18,19,20,21,22,23,24,25,26,27,28,29,30,31,32,33,34,35,36,37,38,39,40,41,42,43,44,45,46,47,48,49,50,51,52,53,54,55,56,57,58,59,60,61,62,63,64,65,66,67,68,69,70,71,72,73,74,75,76,77,78,79,80,81,82,83,84,85,86,87,88,89,90,91,92,93,94,95,96,97,98,99]. In this study, water storage for a period of one month was the cut off value. Several studies used the same aging protocol, observed a significant decrease in bond strength between the ceramic-cement interface, which proved that this time interval is sufficient to promote a degradation of this interface [16].

Regarding in vitro studies, the main zirconia adhesion protocols involve a mechanical conditioning phase and then the application of chemical adhesion promoters. The use of silane is rationally justified where a layer of silica (e.g., silica-coating, glaze on technique) was created [55], while, on polycrystalline zirconia, solutions based on functional monomers are used [47].

Sandblasting is a process that uses the energy released by the impact of alumina particles (Al_2_O_3_), emitted by a high-speed source. The impact involves the erosion of the material with the formation of a rough, clean, and wettable surface [49]. However, sandblasting can also lead to the formation of surface damage, defects, and cracks. Therefore, the mechanical characteristics of zirconia can be compromised [100]. It is advisable to carry out sandblasting according to adequate parameters in relation to pressure, distance from the source, and particle size. Souza [101] recommends to carry out the sandblasting process using small particles (30 µm) with moderate pressure (2.5 bar) in order to avoid material damages. In 2013, Ozcan [102] proposes a protocol for blasting zirconia, with alumina particles with a diameter between 30 and 50 µm, at a pressure between 0.5 and 2.5 bar for a duration of at least 20 s. The blast jet must be positioned 10 mm from the target, and kept in motion, so as not to create defects.

The laser is proposed in zirconia adhesion as a mechanical conditioning technique. The goal is to increase the surface roughness, in order to create a micromechanical interconnection with the resin. Nd: YAG laser is not able to guarantee satisfactory roughness and adhesion values, which also modifies the power set and time of application [80]. Zirconia overheating causes cracks, residual stress, and monoclinic transformation. Regarding Er: YAG laser, a setting of 2 W produces good roughness, like alumina sandblasting treatment, but the surface shows cracks and defects [35]. Laser application with energy intensity of 400 or 600 mJ is associated with material deterioration, while, with lower values (200 mJ), satisfactory adhesion is not obtained [72,79]. An ultra-short pulse laser (Yb: YAG) has been studied for zirconia conditioning. It emits impulses in the order of 6 picoseconds, with a power of 9 W. The shortness of the impulses allows the rapid removal of small amounts of material that have absorbed the energy of the laser by overheating, without considerable mechanical and thermal damage to the rest of the sample. SEM analysis shows a rough surface without surface defects. In terms of µTBS, the laser treatment seems to be superior to tribo-chemical silica-coating and alumina-sandblasting after a month of water storage [34].

Electrical Discharge Machine (EDM) is an unconventional method that leads to erosion of material through electrical impulses in a dielectric medium. In terms of Shear Bond Strength (SBS), the EDM technique obtains good results. However, by SEM analysis, surface cracks can be highlighted [35].

Zirconia is considered to be an inert material. The surface cannot be activated with hydrofluoric acid etching because it does not act on the crystalline component. Anyway, various acid solutions have been proposed to etch zirconia, based on hydrofluoric and nitric acid applied at a temperature of 100 °C. Acid etching of the zirconia surface with these modalities is less effective than Tribological-chemical treatment [52]. Other authors get positive results for some experimental solutions. Xie [71] obtains good results for adhesion protocols involving hot etching and application of 10-MDP primers. In another study [31], an experimental acid solution (800 mL of ethanol, 200 mL of 37% HCl, and 2 g of ferric chloride) is tested. It seems to be able to dissolve the surface of the zirconia and guarantee good adhesion. The solution is applied at a temperature of 100 °C for 1 hour. Xie [22] gets good results by the use of a 40% HF solution. Although these techniques seem, in some cases, able to promote adhesion [22,31,71], it must be evaluated by the possible negative effects of the use of these methods, which are linked mainly to clinical safety [22].

Sandblasting, like other exclusively mechanical treatments, is able to modify the zirconia surface. However, it is essential to associate these treatments with the use of chemical promoters, capable of improving adhesion. Today 10-MDP-based cements and primers are used for this purpose [14]. Primers contain organophosphate monomers, including 10-MDP, 6-MHPA, or 4-META. The 10-MDP presents a terminal functional group with phosphoric acid, which reacts with zirconia and forms P-O-Zr bonds. The other end of the molecule is occupied by a vinyl terminal group, which allows the copolymerization with the resin. These two functional groups are separated by a carbon chain, which is responsible for characteristics such as viscosity, rigidity, hydrophobicity, and solubility. Solutions containing 10-MDP can promote better adhesion than those containing 4-META, MAC-10, or 3-TMSPMA [24,25,40,47,68]. Chemical adhesion increase occurs as well with a self-adhesive composite cement. However, the use of 10- MDP cement alone does not seem to be able to maintain good adhesion levels after thermocycling [46,49,76]. The use of a 10-MDP based primer is able to increase the bond strength both with a self-adhesive composite (based on 10-MDP or other functional monomers) and traditional composite cement [40,44,46,66]. It seems to be important to use a sufficiently fluid cement to benefit from the effects of sandblasting, despite the kind of composite. Regardless of the results obtained by the various studies, the authors agree that thermocycling strongly affects the bond between sandblasted zirconia and 10-MDP-based materials, which puts the long-term reliability of this adhesion protocol at risk [30,37,81].

Tribochemical silica-coating (TBS) is another method used to promote adhesion to zirconia. This is a sandblasting process that is carried out using alumina-particles covered with silica, which impacts against the surface of the ceramic, as well as creates an irregular surface while releasing silica. The presence of this vitreous component allows the use of silane as a coupling agent. It binds both to the composite and to the silica deposited on the zirconia and improves adhesion [55]. TBS is carried out mainly by two methods: the Rocatec system consists of a traditional sandblasting pretreatment, and a subsequent use of silica-coated alumina particles (110 µm). The Cojet system uses coated alumina particles of silica (30 µm) and can be applied by the chair. The size of the particles used for alumina sandblasting (50 µm and 120 µm) or for tribological-chemical treatment (30 µm and 110 µm) does not affect SBS [57]. The use of a primer containing silane and 10-MDP allows the achievement of a better bond between composite and zirconia compared to the application of silane alone [32,38,85]. The silicatization process, with the tribological-chemical method, is not uniform on the surface of the zirconia. Where there are still areas not covered by silica, 10-MDP acts on the surfaces.

TBS appears to be more resistant to thermocycling than other treatments. According to thermodynamic calculations, the bond between silica and silane is more resistant to hydrolysis than the bond between zirconia and 10-MDP [45]. Several studies agree that TBS, followed by the application of silane-containing primer, is more stable than alumina sandblasting followed by the application of 10-MDP-based primers [28,71]. Other authors, on the other hand, obtain good results for adhesion protocols that involve alumina-sandblasting, with adhesion values comparable to TBS [32,58,60].

Sandblasting with feldspathic ceramic powder appears to have promising results in terms of SBS when compared to the use of silica-coated alumina, with a lower t-m transformation rate and stable results after thermocycling [33]. The use of rotary tools, discs, and diamond burs is not suitable for the treatment of zirconia [8,49]. The zirconia hardness involves the use of aggressive techniques, that inevitably lead to cracks and surface damage. 

Zirconia is a polycrystalline ceramic, not conventionally etched with acid [54]. In order to promote adhesion, some authors have studied the possibility of applying, on the zirconia surface, a glassy layer, which is rich in silicon oxides. Zirconia can, thus, be treated like a glass ceramic. It is etched with hydrofluoric acid and the silane is applied as a coupling agent. This molecule has two different functional groups: the -SiOH group binds to the hydroxyl groups of silica coated surface forming a siloxane bond (Si-O-Si) and other functional groups of the silane (>C=C<) bind to the methacrylate of the resin [56]. The thickness occupied by the glass ceramic layer deposited on the inner surface of the zirconia restorations can lead to a marginal misfit. Moreover, some authors focus on the fragility of this vitreous layer that can start surface defects and crack propagation. The application of a glass ceramic coating, subsequently etched with HF, seems to guarantee good adhesion [46,74]. There is a superiority of spray application systems rather than powder/liquid systems with a clinically acceptable marginal misfit (≈10 µm) [52,78,83,85]. Some authors mark a reduction in the bond strength after artificial aging methods, explained by the fact that the glass ceramic layer is not well bound to zirconia. The bond occurs through weak micromechanical interlocking and Van der Waals interactions susceptible to hydrolysis [97].

Silica deposition on zirconia, which allows the use of silane as a coupling agent, is also pursued by Magnetron-sputtering Physical Vapor Deposition (PVD). Sputtering is a technique for realizing thin films, which allows us to deposit both metallic materials and insulating materials on a substrate. This method of SiO_2_ deposition on the zirconia surface does not guarantee adhesion results comparable to those obtained with traditional treatments [63,86].

The silicatization of the zirconia surface is also obtained through “pyrochemical” techniques. The Silano-Pen system, for example, consists of a lighter containing a solution of butane and silane. When the butane is burned, the silane compound decomposes into SiOx-C fragments that adhere to zirconia, which can be silanized. This method is not sufficiently effective to promote a stable and lasting bond to the composite [8,49].

Zirconia can be modified with a technique called Selective Infiltration Etching (SIE): the ceramic is coated with silica-based material, with a thermal expansion coefficient similar to the zirconia one. During the fusion (when the temperature of 960 °C is reached), this material diffuses in the zirconia structure. Then hydrofluoric acid is applied for about 10 minutes in order to dissolve the glass component completely. The surface of the zirconia appears to be irregular [41,54].

With regard to the cementation phase, the main alternative to composites is the use of a traditional glass ionomer cement, or a CVI modified with resin. In terms of adhesion, the composite cements have better results [10,73]. Regarding the class of resin cements, the choice can essentially fall into two categories: traditional cements or self-adhesive cements. With traditional composites, the bond strength is linked to the effectiveness of preliminary treatments. For mechanical treatment and primer association, they are also less viscous, which may favor penetration into surface micro-porosities and resistance over time. Self-adhesive cements can bind to zirconia, but are not able to, alone, maintain stable long-term adhesion, which are more susceptible to hydrolysis. The association of mechanical conditioning and chemical promoters is essential [37,57,67]. Self-adhesive cement composition can be made of different functional monomers. According to some authors, the 10-MDP self-adhesive cements give better adhesion values [79,87]. In other studies, there is no clear superiority of a cement category [58].

The zirconia prosthesis can be contaminated during the clinical phases: blood, saliva, impression materials, and other contaminants can deposit on the material and interfere with the adhesion mechanism [43]. Cements and primers, by the presence of phosphate groups in their structure, interact with the surface of the zirconia. If contaminants are present, sites that could be occupied by the phosphate monomers become inactive [8]. Some treatments such as cleansing with H_2_O, H_2_O_2_, ethanol, or acetone, the application of orthophosphoric acid, ethyl cellulose-based paints, ultrasonic cleaning, or plasma treatment are all ineffective in removing contaminants [13,43,51,53,62]. Sandblasting with Al_2_O_3_ powder is the most effective method for removing contaminants, even though it can weaken the structure of zirconia if carried out several times on the material. Cleansing with NaOCl-solutions or with the cleaning paste Ivoclean (Ivoclar Vivodent, Schaan, Liechtenstein) (sodium hydroxide, ZrO_2_, water, polyethylene glycol, pigments) seem a valid alternative in the consideration of costs and practicality, and the possible deterioration of the zirconia structure [20,51,53]. The effectiveness of Ivoclean, which is composed of an aqueous solution containing zirconium particles, is based on the chemical affinity between the components of the solution and the saliva contaminants [53]. If saliva contamination occurs when zirconia has already been treated with 10-MDP primer, just 20 seconds of water spray rinsing seems to be sufficient to bring the bond strength back to values comparable to the control group, in which no contamination was made [43]. With the application of the primer, the hydrophobic methacrylate terminations of the 10-MDP molecule are exposed on the surface. This involves the creation of a water-repellent surface that reduces the possibility of saliva, composed of 99% water, to wet the ceramic [43]. Furthermore, if phosphoric acid treatment or Ivoclean application are carried out after primer application, the Shear Bond Strength values decrease, likely to remove the coating of MDP either from a chemical interaction, mechanical debridement from the micro-brush, or both [43].

The introduction of translucent zirconia on the market allowed the realization of monolithic prosthetic products. In relation to this, interest is growing in realizing safe and standardized adhesive cementation protocols of zirconia. It is important to observe the differences on the possibility of conditioning, between traditional and translucent zirconia. Only a few recent studies evaluate the possibility of adhesion of this material. Results show that bonding of highly translucent zirconia exhibits behavior similar to that of traditional 3Y-TZP [12,24].

To date, there are still few clinical studies on the realization of Resin Bonded Fixed Dental Prosthesis (RBFDP). Only five articles were found that meet our inclusion criteria and no one involves full zirconia restorations. Two clinical studies [91,92] concern the outcome of posterior inlay-retained fixed dental prosthesis. The results are contrasting. The longevity of the restorations is to be attributed to the modification of the inlay design (palatine and vestibular extension) rather than the effectiveness of adhesive cementation. Other clinical studies, regarding the realization of incisors cantilever resin-bonded fixed dental prostheses, show good clinical longevity [90,93,94].

Regarding the systematic review included, Thammajaruk [103] collected papers only up to 2016. The meta-analysis compares bond strength results from different kinds of tests (micro and macro). Notwithstanding that, the present review partly agrees with their results.

It could also be useful to broaden the search, including the “Scopus” and “Scholar” databases, to have an even wider view on the subject.

Clinical recommendations are difficult to give, for two main reasons such as the small number of clinical studies found in the literature and the difficulty in comparing laboratory studies that evaluate a number of techniques and obtain often conflicting results. Further in vitro studies, that investigate promising techniques and own better homogeneity on the test set-up characteristics, as well as further clinical trials, are needed to have more evidence to support an adhesion protocol with certain predictable results.

## 5. Conclusions

In literature, we find a variety of adhesion protocols, including the use of different zirconia treatment methods, various adhesion media, different tests, and storage times. The results are difficult to compare.

The combination of a mechanical and chemical treatment is essential for good adhesion. Protocols with greater evidence in the literature include sandblasting with silica-coated particles (that allows the association of silane primers) and traditional alumina sandblasting (combined with the use of chemical promoters like 10-MDP-based products). The latter has less evidence of long-term stability. Other methods involving the silicatization of zirconia obtain promising results that must be validated by further studies.

The choice of the composite cement is less relevant. 

Surface contamination has a negative effect on adhesion.

New highly traslucent zirconia shows a similar behavior, in terms of adhesion, to traditional 3Y-TZP.

An adhesion protocol that provides unequivocal results has not yet been identified.

## Figures and Tables

**Table 1:** Inclusion criteria.

Database	PubMed, Medline; Cochrane Library.
**Publication date**	1 January 2013–31 December 2018
**Keywords**	Zirconia, 3Y-TZP, Adhesion, Adhesive cementation, Bonding, Resin, Composite resin, Composite material, Dentin, Enamel.
**Language**	English
**Type of paper**	In vitro studies, clinical articles, systematic reviews.
**Inclusion criteria**	Studies evaluating adhesion between zirconia and composite.
**Exclusion criteria**	In vitro studies: absence of bonding strength evaluation, insufficient aging (TC <5000 or storage <one month), complete crown specimens; Clinical articles: Case Report, Follow up < 5 years, studies on complete crowns.
**Journal category**	All

**Table 2:** PubMed research No. 1, 2 January 2019.

Search	Query	Items Found
**1**	Zirconia OR 3Y-TZP	7020
**2**	Adhesion OR adhesive cementation OR bonding	372,909
**3**	Resin OR composite resin OR composite material	88,815
**4**	1 AND 2 AND 3	652
**5**	Filters: Publication date from 2013/01/01 to 2018/12/31	370
**Final string**		
(((“zirconium oxide”[Supplementary Concept] OR “zirconium oxide”[All Fields] OR “zirconia”[All Fields]) OR 3Y-TZP[All Fields]) AND (((“J Adhes”[Journal] OR “adhesion”[All Fields]) OR ((“adhesives”[Pharmacological Action] OR “adhesives”[MeSH Terms] OR “adhesives”[All Fields] OR “adhesive”[All Fields]) AND (“cementation”[MeSH Terms] OR “cementation”[All Fields]))) OR (“object attachment”[MeSH Terms] OR (“object”[All Fields] AND “attachment”[All Fields]) OR “object attachment”[All Fields] OR “bonding”[All Fields]))) AND (((“resins, plant”[MeSH Terms] OR (“resins”[All Fields] AND “plant”[All Fields]) OR “plant resins”[All Fields] OR “resin”[All Fields]) OR (“composite resins”[MeSH Terms] OR (“composite”[All Fields] AND “resins”[All Fields]) OR “composite resins”[All Fields] OR (“composite”[All Fields] AND “resin”[All Fields]) OR “composite resin”[All Fields])) OR (composite[All Fields] AND material[All Fields])) AND (“2013/01/01”[PDAT]: “2018/12/31”[PDAT])		

**Table 3:** PubMed research No. 2, 2 January 2019.

Search	Query	Items Found
**1**	Zirconia OR 3Y-TZP	7020
**2**	Adhesion OR adhesive cementation OR bonding	372,909
**3**	Dentin OR enamel	57,574
**4**	1 AND 2 AND 3	158
**5**	Filters: Publication date from 1 January, 2013 to 31 December, 2018	77
**Final string**		
(((“zirconium oxide”[Supplementary Concept] OR “zirconium oxide”[All Fields] OR “zirconia”[All Fields]) OR 3Y-TZP[All Fields]) AND (((“J Adhes”[Journal] OR “adhesion”[All Fields]) OR ((“adhesives”[Pharmacological Action] OR “adhesives”[MeSH Terms] OR “adhesives”[All Fields] OR “adhesive”[All Fields]) AND (“cementation”[MeSH Terms] OR “cementation”[All Fields]))) OR (“object attachment”[MeSH Terms] OR (“object”[All Fields] AND “attachment”[All Fields]) OR “object attachment”[All Fields] OR “bonding”[All Fields]))) AND ((“dentin”[MeSH Terms] OR “dentin”[All Fields]) OR (“dental enamel”[MeSH Terms] OR (“dental”[All Fields] AND “enamel”[All Fields]) OR “dental enamel”[All Fields] OR “enamel”[All Fields])) AND (“2013/01/01”[PDAT]: “2018/12/31”[PDAT])		

**Table 4:** Cochrane Library research, 2 January 2019.

Search	Query	Items Found
**1**	Zirconia OR 3Y-TZP	267
**2**	Adhesion OR adhesive cementation OR bonding	12,030
**3**	Resin OR composite resin OR composite material	6317
**4**	1 AND 2 AND 3	52
**5**	Cochrane Library publication date from Jan 2013 to Dec 2018	31
**Final string**		
((zirconia) OR 3Y-TZP):ti,ab,kw AND (((adhesion) OR adhesive cementation) OR bonding):ti,ab,kw AND (((resin) OR composite resin) OR composite material):ti,ab,kw (Word variations have been searched)” with Cochrane Library publication date Between Jan 2013 and Dec 2018 (Word variations have been searched)		

**Table 5:** Cochrane Library research, 2 January 2019.

	Article	Tested Adhesion Techniques	Results
**1**	Yang et al., 2018 [10]	Different kinds of cement (RMGIC, self-adhesive, MDP-free). Primers and universal adhesives with 10-MDP. Preliminary APA preliminary.	RMGIC get worse adhesion results. Better bond strength for MDP primer (or adhesive) with traditional composite cement, than MDP cement alone.
**2**	Thammajaruk et al., 2019 [11]	Ceramic coating technique (DCM hot bond coating) vs. APA.	APA gives better adhesion and more stable long-term results.
**3**	Shimizu et al., 2018 [12]	Mechanical pre-treatment (none, APA, plasma treatment) and chemical pre-treatment (none, 10-MDP primer).	APA improves the bonding strength and the pre-treatment with MDP primer resulted in better adhesion.
**4**	Piest et al., 2018. [13]	Efficacy of plasma treatment for contaminated zirconia (saliva and silicone).	Plasma treatment is expensive and not efficacious, especially for silicone contamination.
**5**	Yang et al., 2018 [14]	Comparison between two kinds of adhesion protocol: one involves APA followed by MDP- free composite cement, others that involve APA followed by MDP containing product (primer or adhesive or cement).	Product containing 10-MDP (primers, adhesives, and cement) improve zirconia adhesion in comparison APA with MDP-free composite cement.
**6**	Moura et al., 2018 [15]	Comparison of three adhesion techniques: -APA+ MDP- composite cement -MDP-primer + MDP-free composite cement (no APA) -APA+ primer with functional monomer + MDP-free composite cement.	The adhesion protocol that involves APA followed the use of MDP-composite cement has worse results.
**7**	Araùjo et al., 2018 [16]	Compare the effectiveness of an MDP-adhesive as a substitute for TBC adhesion protocol.	Mechanical pre-treatment included in the TBC technique is necessary for an adequate adhesion.
**8**	Grasel et al., 2018 [17]	Evaluation of the effectiveness of mechanical pre-treatment (APA). Comparison of different adhesion systems (universal primer and composite cement) after APA.	Mechanical pre-treatment is necessary for improving adhesion. No substantial differences between the adhesion systems.
**9**	Dos Santos et al., 2018 [18]	Effect of incorporation of TiO_2_ nanotubes in a polycrystalline zirconia on bond strength.	The technique tested has no significant effect.
**0**	Dal Piva et al., 2018 [19]	Efficacy of a heat-treatment after TBC protocol.	Heat treatment is not valid in improving adhesion.
**1**	Yoshida et al., 2018 [20]	Cleaning methods for saliva contaminated zirconia (Ivoclean, ADG, etching gel, APA).	ADG and APA are effective cleaning methods on the alumina blasted zirconia.
**2**	Wille et al., 2017 [21]	Effectiveness comparison of “phosphoric acid esters”-based primer and a self-etching primer applied on sandblasted zirconia. Cementation with composite cement.	Phosphoric acid esters primer gets better results.
**3**	Xie et al., 2017 [22]	Different zirconia treatments (APA Al_2_O_3_, 40% HF 30 min, 40% HF 10 min in US bath) and different 10-MDP primers. Composite cement.	No differences emerge on the type of primer, nor on the way the acid is applied. Treatment with HF achieves results comparable to sandblasting.
**4**	Pitta et al., 2017 [23]	The study evaluates the effect of saliva contamination on the effectiveness of the adhesive system.	Some adhesive systems do not appear to be affected by saliva contamination.
**5**	Yagawa et al., 2018 [24]	Comparison of some primers containing different adhesive monomers. Cementation with self-curing or dual composite.	Dual cement ensures better adhesion. Major SBS for samples treated with 10-MDP primer.
**6**	Noda et al., 2017 [25]	Comparison of different primers with functional monomers.	Primer containing 10-MDP exhibits higher bond strength than MAC-10 primer.
**7**	Chuang et al., 2017 [26]	Comparison of silane, 10-MDP, or both MDP and silane primers on sandblasted samples.	10-MDP primers get better SBS.
**8**	Elsayed et al., 2017 [27]	Sandblasted samples, subjected to the application of different primer/composite cement adhesive systems.	-APA + Monobond Plus (silane/adhesive monomers) + Variolink Esthetic DC. -APA + All Bond universal (10-MDP) + Duo Link Universal.
**9**	Galvão Ribeiro et al., 2018 [28]	Comparison between APA and TBC treatment followed by application of silane or silane/10-MDP primers. Self-adhesive composite.	TBC + silane/10-MDP primer + self-adhesive composite.
**0**	Chen C et al., 2017 [29]	Effect of storage in aqueous solutions (acid, basic, or neutral) on adhesion. Sandblasted samples, treated with two different MDP primers, cemented with a composite.	Values of SBS greater for samples deposited in alkaline solution.
**1**	Tsujimoto e al., 2017 [30]	Bond durability of universal adhesives.	Thermocycling decreases bond strength.
**2**	Sakrana and Ozcan, 2017 [31]	Different mechanical treatments (APA, CH_2_Cl_2_, HCl).	Better adhesion for HCl e APA.
**3**	Akazawa et al., 2017 [32]	Comparison between APA and TBC followed by the application of different primers. MDP-free composite cement.	-TBC + silane/10-MDP primer. -SAPA Al_2_O_3_ (50-70 µm) + silano/10-MDP primer.
**4**	Wandscher et al., 2016 [33]	Sandblasting with leucite powder, feldspar ceramic or Cojet method. Silane and adhesive cement application.	Better results for leucite powder sandblasting.
**5**	Esteves-Oliveira et al., 2016 [34]	Comparison between APA, TBC, ultrashort pulses laser. Self-adhesive composite.	Laser treatment is the more effective one.
**6**	Rona et al., 2017 [35]	Comparison between APA, TBS, Er: YAG e EDM (Electric Discharge Machine). MDP/silane or silane primer; MDP- based composite.	Better SBS values for EDM e Rocatec.
**7**	Sawada et al., 2016 [36]	Effectiveness of experimental conditioners, based on silica and quartz, applied before sintering.	Experimental solution does not improve significantly adhesion.
**8**	Zhao et al., 2016 [37]	Comparison of different primer/cement systems in improving zirconia adhesion.	Using an MDP-primer before cement improves adhesion, regardless of the type of cement (self-adhesive or MDP-free).
**9**	Iwasaki et al., 2016 [38]	Zirconia treatment with APA or TBC, followed by primer application with different functional components and traditional composite cement.	-TBC + 10-MDP/silane primer + traditional composite cement.
**0**	Passia et al., 2016 [39]	Effectiveness of different primers and composite cements after APA Al_2_O_3_.	-APA Al_2_O_3_ associated with MDP cement or phosphoric acid methacrylate cement and MDP primer.
**1**	Lopes et al., 2016 [40]	Different kinds of primers on sandblasted zirconia. MDP free cement.	MDP-based primers improve adhesion.
**2**	Salem et al., 2016 [41]	Different kind of treatments (APA Al_2_O_3_, SIE, “Modified fusion sputtering”). Self-adhesive composite.	-SIE or “Modified fusion sputtering” + silane/10-MDP primer.
**3**	Hallmann et al., 2016 [42]	Mechanical pre-treatments (APA with alumina or zirconia, abrasive paper, acid solution, plasma, argon-ion bombardment); 10-MDP composite.	The most effective method is APA with Al_2_O_3_. Increased adhesion strength even with sandblasting with zirconia particles, which seems to be less harmful.
**4**	Angkasith et al., 2016 [43]	Effect of saliva contamination with the use of 10-MDP primers.	If the contamination occurs after the primer, rinsing with water is sufficient. Otherwise, Ivoclean and APA are effective.
**5**	Bomicke et al., 2016 [44]	Comparison between different mechanical treatments (APA, Cojet, and Rocatec TBC), and comparison between the adhesive system.	-Rocatec + silane/10-MDP primer + 10-MDP composite.
**6**	Xie et al., 2016 [45]	Comparison between TBC and APA with different MDP primers.	-APA + Z-Prime plus+ 10-MDP primer -TBC
**7**	Cheung et al., 2015 [46]	Comparison of different surface treatments (vitrification, APA) followed by the application or not of silane/MDP primers and cementation with an MDP composite.	Liner (pre sintering) + HF + silane/MDP primer.
**8**	Ahn et al., 2015 [47]	Comparison between sandblasted or not zirconia. Application or not of primers with 10-MDP or other adhesive monomers. 10-MDP cement.	Good adhesion for APA + Primer 10-MDP + 10-MDP cement. Self-adhesive cement without preliminary sandblasting does not guarantee adhesion.
**9**	Alves et al., 2016 [48]	Comparison on cement (traditional composite or self-adhesive), and different substrates (Cojet, Rocatec, silane primer/10-MDP).	Better SBS for primer + traditional composite.
**0**	Yenisey et al., 2016 [49]	Effectiveness of various surface treatments and their association (APA, Cojet, Rocatec, Er: YAG, silane primer, Silano-Pen).	-APA + Cojet + silane.
**1**	Pereira et al., 2015 [50]	Comparison of application of various types of primers with or without sandblasting.	In general, sandblasting increases the bond strength if associated with the use of the primer, except for Scotchbond Universal (universal primer) and MZ Primer (primer with adhesive monomers).
**2**	Kim DH et al., 2015 [51]	Different cleaning methods: NaOCl, APA, Ivoclean, H_2_O_2_, H_2_O, and sodium dodecyl sulfate.	Effective for saliva cleansing NaOCl, Ivoclean, and sandblasting.
**3**	Liu D et al., 2015 [52]	TBC comparison with application of acid solutions (Nitric and Fluoridric acid) and application of pre-sintering silica particles. Silane/10-MDP primer, 10-MDP composite.	TBC method and silica particle deposition have higher SBS values.
**4**	Ishii et al., 2015 [53]	Comparison of saliva cleansing methods: water, sandblasting, Ivoclean, orthophosphoric acid.	Sandblasting and Ivoclean are effective.
**5**	Jiang et al., 2014 [54]	APA Al_2_O_3_ vs. SIE. 4-META-based composite.	Both methods increase adhesion values when compared to the control.
**6**	Oliveira-Ogliari et al., 2015 [55]	Effectiveness of solutions based on zirconia precursors compared with TBC. Silane, adhesive cement.	Promising results for experimental solutions.
**7**	Lung et al., 2015 [56]	Comparison of a solution based on silicon nitride with TBC. Silane, adhesive cement.	TBC gets better results.
**8**	Sciasci et al., 2015 [57]	Different surface treatments (APA, TBC) in association with different types of cement (modified CVI and adhesive cements).	High adhesion values for TBC with traditional adhesive cements or self-adhesive.
**9**	Qeblawi et al., 2015 [58]	Comparison of zirconia treatment (APA and TBC) and adhesive cement type.	-TBC (Cojet) + silane + self-adhesive.-APA (Al_2_O_3_ 50 µm) + self-adhesive (MDP).
**0**	Feitosa et al., 2015 [59]	Different saliva cleansing methods: water, Ivoclean, orthophosphoric acid, isopropanol.	Ivoclean is the most effective of the tested methods.
**1**	Yi et al., 2015 [60]	APA and TBC, followed by primer application with different functional components and cementation with 10-MDP composite.	-APA Al_2_O_3_ + 10-MDP primer + 10-MDP composite.
**2**	Kim JH et al., 2015 [61]	Effectiveness comparison of various 10-MDP based primers. For this purpose, no preliminary treatments are done on zirconia and a traditional composite cement is used.	Primer universali All Bond Universal (10-MDP) and Single Bond Universal (10-MDP/silane) get better results than the Alloy Primer (10-MDP).
**3**	Klosa et al., 2014 [62]	Effectiveness of a solution of ethyl cellulose in the removal of contaminants.	The experimental solution improves SBS but does not reach the values of the uncontaminated sample.
**4**	Druck et al., 2015 [63]	Comparison of deposition of silica nanofilm (magnetron sputtering PVD) with tribological-chemical treatment. Silane and adhesive cement application.	Similar results (TBS) for TBC and Si nanofilm (5 nm).
**5**	De Souza et al., 2014 [64]	Different primers for zirconia, adhesive systems, and MDP- based cements.	Better adhesion values for samples in which the primer is applied.
**6**	Chen C et al., 2014 [65]	Comparison between TBC and APA followed or not by application of primer (Z-Prime Plus), both with traditional composite cement and self-adhesive (RelyX Unicem).	-TBC+ silane + traditional composite cement.
**7**	Shin et al., 2014 [66]	Two different MDP composites on zirconia treated with various methods (MDP primer, APA + primer, Cojet).	No significant differences on the type of cement. Best SBS for APA followed by the application of the 10-MDP primer.
**8**	Da Silva et al., 2014 [67]	Comparison of zirconia treatment (10-MDP primer vs. TBC) and comparison of cement type (traditional composite cement and self-adhesive, with adhesive monomers).	Best result for self-adhesive composite, in association with tribological-chemical treatment.
**9**	Oba et al., 2014 [68]	Efficacy of different primers on sandblasted zirconia.	MDP primers get better results, and are indifferent if silane is also present.
**0**	Liu et al., 2014 [69]	Comparison between: Rocatec, Glazing Porcelain + HF, pre-sintering silica powder application, pre-sintering zirconia powder application. Composite self-adhesive.	High SBS values for tTBC treatment and zirconia powder.
**1**	Erdem et al. 2014 [70]	Comparison of zirconia treatments (APA, TBC, Er: YAG), associated with different cements.	-Air abrasion 110 µm + self-adhesive composite.-Rocatec + silane + both traditional or self-adhesive cement.
**2**	Xie et al., 2013 [71]	Comparison of different treatments of zirconia (APA, Cojet, acid etching), followed by application or not of the primer.	-TBC (Cojet) + silane + MDP-free composite. -Hot etching + MDP primer + MDP-free composite.
**3**	Lin et al., 2013 [72]	Comparison of different treatments of zirconia (sandblasting with Al_2_O_3_ and Er: YAG laser).	The use of the Er: YAG laser is not able to increase the adhesion values.
**4**	Turker et al., 2013 [73]	Comparison of adhesion of CVI, CVI modified with resin, and MDP composite cements. Preliminary blasting.	Better adhesion values for self-adhesive cements.
**5**	Cheung et al., 2014 [74]	Comparison of different surface treatments (vitrification, APA) followed by the application or not of silane/MDP primers, cementation with MDP composite.	-TBC + silane/MDP + MDP cement.-Vitrification + HF+ silane/MDP+ MDP cement.
**6**	Keul et al., 2013 [75]	Comparison of the use of self-adhesive cements alone or in combination with primers containing adhesive monomers.	The use of the primer improves the bond strength.
**7**	Sarmento et al., 2014 [76]	APA and TBC comparison. Silane/10-MDP primer and 10-MDP composite.	After thermocycling spontaneous de-cementation of all the samples.
**8**	Heikkinen et al., 2013 [77]	Effect of different kind of silane on silica-coated alumina blasted zirconia.	Not significant differences.
**9**	Bottino et al., 2014 [78]	Comparison of two surface treatments of zirconia (vitrification and TB) associated with two different 10-MDP based resin cements.	Panavia F cement guarantees better adhesion, in particular in association with vitrification.
**0**	Gomes et al., 2015 [79]	Confronto trattamento zirconia (TBC, Laser Er: YAG) e tipologia cemento (cemento 10-MDP e cemento autoadesivo con altri monomeri).	-TBC (Rocatec) + silane + 10-MDP composite.
**1**	Liu L et al., 2015 [80]	Different zirconnia treatments (APA Al_2_O_3_, Nd: YAG laser). MDP-based cement.	-APA Al_2_O_3_ + MDP cement.
**2**	Seto et al., 2013 [81]	Comparison of different types of adhesive cement on sandblasted samples.	Higher adhesion values for cement with 10-MDP (Panavia 2.0 + Oxiguard primer) and GCem (self-adhesive with other monomers).
**3**	Baldissara et al., 2013 [82]	Comparison TBC with ceramic liner, and between self-adhesive composite (Panavia F e Rely X).	TBC achieves superior bond strength, especially in association with RelyX. Panavia F gives better results in association with the liner.
**4**	Vanderlei et al., 2014 [83]	Comparison between “glaze on technique and TBC.” MDP composite cement.	-Low fusing porcelain glaze + HF + silane MDPcomposite.
**5**	Wang et al., 2014 [84]	Use of MDP-primers (with different air-dried pressure) on sandblasted zirconia.	The pressure can affect the result depending on the primer used.
**6**	Saker et al., 2013 [85]	Comparison of different treatments (APA, TBC + silane or 10-MDP based primer, “glaze on” technique). Cementation with MDP composite.	-TBC + MDP primer - “Glaze on” technique + HF + silane.
**7**	Queiroz et al., 2013 [86]	Comparison of different zirconia treatments (sandblasting + primer, only primer, silica nanofilm with magnetron sputtering) and different cements (10-MDP, HEMA, other monomers).	-Air abrasion (Al_2_O_3_ 45µm) + Metal/zirconia primer + self-adhesive composite.
**8**	De Sà Barbosa et al., 2013 [87]	Effectiveness comparison of some self-adhesive composite cements containing adhesive monomers other than 10-MDP (RelyX Unicem, BisCem, G-Cem, SeT) with traditional composite cement (RelyX ARC). APA 50 µm.	The only group to maintain higher values after 1 year is the one cemented with G-Cem.
**9**	Lung et al., 2013 [88]	Comparison between TEOS sol-gel technique and TBC.	Silica coating method improved adhesion more effectively.
**0**	Subasi et al., 2014 [89]	Comparison between mechanical treatments (APA Al_2_O_3_, TBC, Er: YAG laser) and between the cement (MDP or other monomers-based).	No differences between APA and TBC, with better results for MDP cements.
RMGIC, Resin Modified Glass Ionomer Cement. APA, Air Particle Abrasion. TBC, Tribochemical silica coating. SBS, Shear Bond Strength. TBS, Tensile Bond Strength.			

**Table 6:** Clinical studies.

	Article	Type of Restoration	Adhesion Protocol	Follow-Up	Overall Survival Rate
**1**	Kern et al., 2017 [90]	Single-retainer RBFDP	-APA Al_2_O_3_ 50 µm, 10-MDP self-adhesive cement -Zirconia primer MDP-free composite cement.	10 years	98.2%
**2**	Rathmann et al., 2017 [91]	IRFDP	Tribochemical silica coating, silane, 10-MDP self-adhesive cement or MDP-free.	5 years	21.2%
**3**	Chaar et Kern, 2015 [92]	IRFDP	APA Al_2_O_3_ 50 µm, self-adhesive 10-MDP cement.	5 years	95.8%
**4**	Sasse et Kern, 2014 [93]	Single-retainer RBFDP	APA Al_2_O_3_ 50 µm, self-adhesive 10-MDP cement.	6 years	91.1%
**5**	Sasse et Kern, 2013 [94]	Single-retainer RBFDP	-APA Al_2_O_3_ 50 µm, 10-MDP self-adhesive cement -Zirconia primer MDP-free composite cement.	5 years	89.4%
RBFDP, Resin Bonded Fixed Dental Prosthesis. IRFDP, Inlay Retained Fixed Dental Prosthesis. APA, Air Particle Abrasion.					

**Table 7:** Systematic review discarded after full-text examination.

	Article	Cause for Exclusion
**1**	Blatz et al., 2017 [95]	Review of clinical trials, includes studies on complete crowns.
**2**	Khan et al., 2017 [9]	Review of laboratory studies, do not consider the aging factor.
**3**	Tzanakakis et al., 2016 [8]	Review of laboratory studies, do not consider the aging factor.
**4**	Luthra et kaur, 2016 [96]	Review of laboratory studies, do not consider the aging factor.
**5**	Ozcan et Bernasconi, 2015 [7]	Review of laboratory studies, do not consider the aging factor.
**6**	Inokoshi et al., 2014 [97]	Review of laboratory studies, does not observe the inclusion criteria relating to aging, and sets an “aging” limit at 1000 TC.
**7**	Miyazaki et al., 2013 [2]	Review of laboratory and clinical studies, do not consider the aging factor, and includes studies on complete crowns.

## References

[1] Colombo M., Poggio C., Lasagna A., Chiesa M., Scribante A. (2019). Vickers micro-hardness of new restorative CAD/CAM dental materials: Evaluation and comparison after exposure to acidic drink. Materials (Basel).

[2] Miyazaki T., Nakamura T., Matsumura H., Ban S., Kobayashi T. (2013). Current status of zirconia restoration. J. Prosthodont. Res..

[3] Zhang Y., Lawn B.R. (2018). Novel Zirconia Materials in Dentistry. J. Dent. Res..

[4] Shahmiri R., Standard O.C., Hart J.N., Sorrell C.C. (2018). Optical properties of zirconia ceramics for esthetic dental restorations: A systematic review. J. Prosthet. Dent..

[5] Lee J.H., Kim S.H., Han J.S., Yeo I.L., Yoon H.I. (2019). Optical and Surface Properties of Monolithic Zirconia after Simulated Toothbrushing. Materials (Basel).

[6] Carrabba M., Keeling A.J., Aziz A., Vichi A., Fabian Fonzar R., Wood D. (2017). Translucent zirconia in the ceramic scenario for monolithic restorations: A flexural strength and translucency comparison test. J. Dent..

[7] Ozcan M., Bernasconi M. (2015). Adhesion to zirconia used for dental restorations: A systematic review and meta-analysis. J. Adhes Dent..

[8] Tzanakakis E.G., Tzoutzas I.G., Koidis P.T. (2016). Is there a potential for durable adhesion to zirconia restorations? A systematic review. J. Prosthet. Dent..

[9] Khan A.A., Al Kheraif A.A., Jamaluddin S., Elsharawy M., Divakar D.D. (2017). Recent Trends in Surface Treatment Methods for Bonding Composite Cement to Zirconia: A Reveiw. J. Adhes Dent..

[10] Yang L., Xie H., Meng H., Wu X., Chen Y., Zhang H. (2018). Effects of Luting Cements and Surface Conditioning on Composite Bonding Performance to Zirconia. J. Adhes Dent..

[11] Thammajaruk P., Buranadham S., Thanatvarakorn O., Ferrari M., Guazzato M. (2019). Influence of glass-ceramic coating on composite zirconia bonding and its characterization. Dent. Mater..

[12] Shimizu H., Inokoshi M., Takagaki T., Uo M., Minakuchi S. (2018). Bonding Efficacy of 4-META/MMA-TBB Resin to Surface-treated Highly Translucent Dental Zirconia. J. Adhes Dent..

[13] Piest C., Wille S., Strunskus T., Polonskyi O., Kern M. (2018). Efficacy of Plasma Treatment for Decontaminating Zirconia. J. Adhes Dent..

[14] Yang L., Chen B., Xie H., Chen Y., Chen Y., Chen C. (2018). Durability of Resin Bonding to Zirconia Using Products Containing 10-Methacryloyloxydecyl Dihydrogen Phosphate. J. Adhes Dent..

[15] Moura D.M.D., do Nascimento Januario A.B., de Araujo A.M.M., de Oliveira Dal Piva A.M., Ozcan M., Bottino M.A., Souza R.O.A. (2018). Effect of primer-cement systems with different functional phosphate monomers on the adhesion of zirconia to dentin. J. Mech. Behav. Biomed. Mater..

[16] Araujo A.M.M., Januario A., Moura D.M.D., Tribst J.P.M., Ozcan M., Souza R.O.A. (2018). Can the Application of Multi-Mode Adhesive be a Substitute to Silicatized/Silanized Y-TZP Ceramics?. Braz. Dent. J..

[17] Grasel R., Santos M.J., Rego H.C., Rippe M.P., Valandro L.F. (2018). Effect of Resin Luting Systems and Alumina Particle Air Abrasion on Bond Strength to Zirconia. Oper. Dent..

[18] Dos Santos A.F., Sandes de Lucena F., Sanches Borges A.F., Lisboa-Filho P.N., Furuse A.Y. (2018). Incorporation of TiO2 nanotubes in a polycrystalline zirconia: Synthesis of nanotubes, surface characterization, and bond strength. J. Prosthet. Dent..

[19] Dal Piva A.M.O., Carvalho R.L.A., Lima A.L., Bottino M.A., Melo R.M., Valandro L.F. (2019). Silica coating followed by heat-treatment of MDP-primer for resin bond stability to yttria-stabilized zirconia polycrystals. J. Biomed. Mater. Res. B Appl. Biomater..

[20] Yoshida K. (2018). Influence of cleaning methods on resin bonding to saliva-contaminated zirconia. J. Esthet. Restor. Dent..

[21] Wille S., Lehmann F., Kern M. (2017). Durability of Resin Bonding to Lithium Disilicate and Zirconia Ceramic using a Self-etching Primer. J. Adhes Dent..

[22] Xie H., Cheng Y., Chen Y., Qian M., Xia Y., Chen C. (2017). Improvement in the Bonding of Y-TZP by Room-temperature Ultrasonic HF Etching. J. Adhes Dent..

[23] Pitta J., Branco T.C., Portugal J. (2018). Effect of saliva contamination and artificial aging on different primer/cement systems bonded to zirconia. J. Prosthet. Dent..

[24] Yagawa S., Komine F., Fushiki R., Kubochi K., Kimura F., Matsumura H. (2018). Effect of priming agents on shear bond strengths of resin-based luting agents to a translucent zirconia material. J. Prosthodont. Res..

[25] Noda Y., Nakajima M., Takahashi M., Mamanee T., Hosaka K., Takagaki T., Ikeda M., Foxton R.M., Tagami J. (2017). The effect of five kinds of surface treatment agents on the bond strength to various ceramics with thermocycle aging. Dent. Mater. J..

[26] Chuang S.F., Kang L.L., Liu Y.C., Lin J.C., Wang C.C., Chen H.M., Tai C.K. (2017). Effects of silane- and MDP-based primers application orders on zirconia-resin adhesion-A ToF-SIMS study. Dent. Mater..

[27] Elsayed A., Younes F., Lehmann F., Kern M. (2017). Tensile Bond Strength of So-called Universal Primers and Universal Multimode Adhesives to Zirconia and Lithium Disilicate Ceramics. J. Adhes Dent..

[28] Galvao Ribeiro B.R., Galvao Rabelo Caldas M.R., Almeida A.A., Fonseca R.G., Adabo G.L. (2018). Effect of surface treatments on repair with composite resin of a partially monoclinic phase transformed yttrium-stabilized tetragonal zirconia. J. Prosthet. Dent..

[29] Chen C., Chen Y., Lu Z., Qian M., Xie H., Tay F.R. (2017). The effects of water on degradation of the zirconia-resin bond. J. Dent..

[30] Tsujimoto A., Barkmeier W.W., Takamizawa T., Wilwerding T.M., Latta M.A., Miyazaki M. (2017). Interfacial Characteristics and Bond Durability of Universal Adhesive to Various Substrates. Oper. Dent..

[31] Sakrana A.A., Ozcan M. (2017). Effect of chemical etching solutions versus air abrasion on the adhesion of self-adhesive resin cement to IPS e.max ZirCAD with and without aging. Int. J. Esthet. Dent..

[32] Akazawa N., Koizumi H., Nogawa H., Nakayama D., Kodaira A., Matsumura H. (2017). Effect of mechanochemical surface preparation on bonding to zirconia of a tri-n-butylborane initiated resin. Dent. Mater. J..

[33] Wandscher V.F., Fraga S., Pozzobon J.L., Soares F.Z., Foletto E.L., May L.G., Valandro L.F. (2016). Tribochemical Glass Ceramic Coating as a New Approach for Resin Adhesion to Zirconia. J. Adhes Dent..

[34] Esteves-Oliveira M., Jansen P., Wehner M., Dohrn A., Bello-Silva M.S., Eduardo C.P., Meyer-Lueckel H. (2016). Surface Characterization and Short-term Adhesion to Zirconia after Ultra-short Pulsed Laser Irradiation. J. Adhes Dent..

[35] Rona N., Yenisey M., Kucukturk G., Gurun H., Cogun C., Esen Z. (2017). Effect of electrical discharge machining on dental Y-TZP ceramic-resin bonding. J. Prosthodont. Res..

[36] Sawada T., Spintzyk S., Schille C., Zöldföldi J., Paterakis A., Schweizer E., Ingrid S., Frank R., Jurgen G.G. (2016). Influence of Pre-Sintered Zirconia Surface Conditioning on Shear Bond Strength to Resin Cement. Materials (Basel).

[37] Zhao L., Jian Y.T., Wang X.D., Zhao K. (2016). Bond strength of primer/cement systems to zirconia subjected to artificial aging. J. Prosthet. Dent..

[38] Iwasaki T., Komine F., Fushiki R., Kubochi K., Shinohara M., Matsumura H. (2016). Shear bond strengths of an indirect composite layering material to a tribochemically silica-coated zirconia framework material. Dent. Mater. J..

[39] Passia N., Mitsias M., Lehmann F., Kern M. (2016). Bond strength of a new generation of universal bonding systems to zirconia ceramic. J. Mech. Behav. Biomed. Mater..

[40] Lopes G.C., Spohr A.M., De Souza G.M. (2016). Different Strategies to Bond Bis-GMA-based Resin Cement to Zirconia. J. Adhes Dent..

[41] Salem R., Naggar G.E., Aboushelib M., Selim D. (2016). Microtensile Bond Strength of Resin-bonded Hightranslucency Zirconia Using Different Surface Treatments. J. Adhes Dent..

[42] Hallmann L., Ulmer P., Lehmann F., Wille S., Polonskyi O., Johannes M., Köbel S., Trottenberg T., Bornholdt S., Haase F. (2016). Effect of surface modifications on the bond strength of zirconia ceramic with resin cement resin. Dent. Mater..

[43] Angkasith P., Burgess J.O., Bottino M.C., Lawson N.C. (2016). Cleaning Methods for Zirconia Following Salivary Contamination. J. Prosthodont.

[44] Bomicke W., Schurz A., Krisam J., Rammelsberg P., Rues S. (2018). Durability of Resin-Zirconia Bonds Produced Using Methods Available in Dental Practice. J. Adhes Dent..

[45] Xie H., Li Q., Zhang F., Lu Y., Tay F.R., Qian M., Chen C. (2016). Comparison of resin bonding improvements to zirconia between one-bottle universal adhesives and tribochemical silica coating, which is better?. Dent. Mater..

[46] Cheung G.J., Botelho M.G. (2015). Zirconia Surface Treatments for Resin Bonding. J. Adhes Dent..

[47] Ahn J.S., Yi Y.A., Lee Y., Seo D.G. (2015). Shear Bond Strength of MDP-Containing Self-Adhesive Resin Cement and Y-TZP Ceramics: Effect of Phosphate Monomer-Containing Primers. Biomed. Res. Int..

[48] Alves M., Campos F., Bergoli C.D., Bottino M.A., Ozcan M., Souza R. (2016). Effect of Adhesive Cementation Strategies on the Bonding of Y-TZP to Human Dentin. Oper. Dent..

[49] Yenisey M., Dede D.O., Rona N. (2016). Effect of surface treatments on the bond strength between resin cement and differently sintered zirconium-oxide ceramics. J. Prosthodont. Res..

[50] Pereira Lde L., Campos F., Dal Piva A.M., Gondim L.D., Souza R.O., Ozcan M. (2015). Can application of universal primers alone be a substitute for airborne-particle abrasion to improve adhesion of resin cement to zirconia?. J. Adhes Dent..

[51] Kim D.H., Son J.S., Jeong S.H., Kim Y.K., Kim K.H., Kwon T.Y. (2015). Efficacy of various cleaning solutions on saliva-contaminated zirconia for improved resin bonding. J. Adv. Prosthodont..

[52] Liu D., Tsoi J.K., Matinlinna J.P., Wong H.M. (2015). Effects of some chemical surface modifications on resin zirconia adhesion. J. Mech. Behav. Biomed. Mater..

[53] Ishii R., Tsujimoto A., Takamizawa T., Tsubota K., Suzuki T., Shimamura Y., Miyazaki M. (2015). Influence of surface treatment of contaminated zirconia on surface free energy and resin cement bonding. Dent. Mater. J..

[54] Jiang T., Chen C., Lv P. (2014). Selective infiltrated etching to surface treat zirconia using a modified glass agent. Adhes Dent..

[55] Oliveira-Ogliari A., Collares F.M., Feitosa V.P., Sauro S., Ogliari F.A., Moraes R.R. (2015). Methacrylate bonding to zirconia by in situ silica nanoparticle surface deposition. Dent. Mater..

[56] Lung C.Y., Liu D., Matinlinna J.P. (2015). Silica coating of zirconia by silicon nitride hydrolysis on adhesion promotion of resin to zirconia. Mater. Sci. Eng. C Mater. Biol. Appl..

[57] Sciasci P., Abi-Rached F.O., Adabo G.L., Baldissara P., Fonseca R.G. (2015). Effect of surface treatments on the shear bond strength of luting cements to Y-TZP ceramic. J. Prosthet. Dent..

[58] Qeblawi D.M., Campillo-Funollet M., Munoz C.A. (2015). In vitro shear bond strength of two self-adhesive resin cements to zirconia. J Prosthet. Dent..

[59] Feitosa S.A., Patel D., Borges A.L., Alshehri E.Z., Bottino M.A., Ozcan M., Valandro L.F., Bottino M.C. (2015). Effect of cleansing methods on saliva-contaminated zirconia--an evaluation of resin bond durability. Oper. Dent..

[60] Yi Y.A., Ahn J.S., Park Y.J., Jun S.H., Lee I.B., Cho B.H., Son H.H., Seo D.G. (2015). The effect of sandblasting and different primers on shear bond strength between yttria-tetragonal zirconia polycrystal ceramic and a self-adhesive resin cement. Oper. Dent..

[61] Kim J.H., Chae S.Y., Lee Y., Han G.J., Cho B.H. (2015). Effects of multipurpose, universal adhesives on resin bonding to zirconia ceramic. Oper. Dent..

[62] Klosa K., Warnecke H., Kern M. (2014). Effectiveness of protecting a zirconia bonding surface against contaminations using a newly developed protective lacquer. Dent. Mater..

[63] Druck C.C., Pozzobon J.L., Callegari G.L., Dorneles L.S., Valandro L.F. (2015). Adhesion to Y-TZP ceramic: Study of silica nanofilm coating on the surface of Y-TZP. J. Biomed. Mater. Res. B Appl. Biomater..

[64] De Souza G., Hennig D., Aggarwal A., Tam L.E. (2014). The use of MDP-based materials for bonding to zirconia. J. Prosthet. Dent..

[65] Chen C., Xie H., Song X., Burrow M.F., Chen G., Zhang F. (2014). Evaluation of a commercial primer for bonding of zirconia to two different resin composite cements. J. Adhes Dent..

[66] Shin Y.J., Shin Y., Yi Y.A., Kim J., Lee I.B., Cho B.H., Son H.H., Seo D.G. (2014). Evaluation of the shear bond strength of resin cement to Y-TZP ceramic after different surface treatments. Scanning.

[67] Da Silva E.M., Miragaya L., Sabrosa C.E., Maia L.C. (2014). Stability of the bond between two resin cements and an yttria-stabilized zirconia ceramic after six months of aging in water. J. Prosthet. Dent..

[68] Oba Y., Koizumi H., Nakayama D., Ishii T., Akazawa N., Matsumura H. (2014). Effect of silane and phosphate primers on the adhesive performance of a tri-n-butylborane initiated luting agent bonded to zirconia. Dent. Mater. J..

[69] Liu D., Pow E.H.N., Tsoi J.K., Matinlinna J.P. (2014). Evaluation of four surface coating treatments for resin to zirconia bonding. J. Mech. Behav. Biomed. Mater..

[70] Erdem A., Akar G.C., Erdem A., Kose T. (2014). Effects of different surface treatments on bond strength between resin cements and zirconia ceramics. Oper. Dent..

[71] Xie H., Chen C., Dai W., Chen G., Zhang F. (2013). In vitro short-term bonding performance of zirconia treated with hot acid etching and primer conditioning etching and primer conditioning. Dent. Mater. J..

[72] Lin Y., Song X., Chen Y., Zhu Q., Zhang W. (2013). Effect of Er:YAG laser irradiation on bonding property of zirconia ceramics to resin cement. Photomed. Laser Surg..

[73] Turker S.B., Ozcan M., Mandali G., Damla I., Bugurman B., Valandro L.F. (2013). Bond strength and stability of 3 luting systems on a zirconia-dentin complex. Gen. Dent..

[74] Cheung G.C., Botelho M.G., Matinlinna J.P. (2014). Effect of surface treatments of zirconia ceramics on the bond strength to resin cement. J. Adhes Dent..

[75] Keul C., Liebermann A., Roos M., Uhrenbacher J., Stawarczyk B., Ing D. (2013). The effect of ceramic primer on shear bond strength of resin composite cement to zirconia: A function of water storage and thermal cycling. J. Am. Dent. Assoc..

[76] Sarmento H.R., Campos F., Sousa R.S., Machado J.P., Souza R.O., Bottino M.A., Ozcan M. (2014). Influence of air-particle deposition protocols on the surface topography and adhesion of resin cement to zirconia. Acta Odontol. Scand..

[77] Heikkinen T.T., Matinlinna J.P., Vallittu P.K., Lassila L.V. (2013). Long term water storage deteriorates bonding of composite resin to alumina and zirconia short communication. Open. Dent. J..

[78] Bottino M.A., Bergoli C., Lima E.G., Marocho S.M., Souza R.O., Valandro L.F. (2014). Bonding of Y-TZP to dentin: Effects of Y-TZP surface conditioning, resin cement type, and aging. Oper. Dent..

[79] Gomes A.L., Ramos J.C., Santos-del Riego S., Montero J., Albaladejo A. (2015). Thermocycling effect on microshear bond strength to zirconia ceramic using Er:YAG and tribochemical silica coating as surface conditioning. Lasers Med. Sci..

[80] Liu L., Liu S., Song X., Zhu Q., Zhang W. (2015). Effect of Nd: YAG laser irradiation on surface properties and bond strength of zirconia ceramics. Lasers Med. Sci..

[81] Seto K.B., McLaren E.A., Caputo A.A., White S.N. (2013). Fatigue behavior of the resinous cement to zirconia bond. Oper Dent..

[82] Baldissara P., Querzè M., Monaco C., Scotti R., Fonseca R.G. (2013). Efficacy of surface treatments on the bond strength of resin cements to two brands of zirconia ceramic. J. Adhes Dent..

[83] Vanderlei A., Bottino M.A., Valandro L.F. (2014). Evaluation of resin bond strength to yttria-stabilized tetragonal zirconia and framework marginal fit: Comparison of different surface conditionings. Oper. Dent..

[84] Wang C., Niu L.N., Wang Y.J., Jiao K., Liu Y., Zhou W., Shen L.J., Fang M., Li M., Zhang X. (2014). Bonding of resin cement to zirconia with high pressure primer coating. PLoS ONE.

[85] Saker S., Ibrahim F., Ozcan M. (2013). Effect of different surface treatments on adhesion of In-Ceram Zirconia to enamel and dentin substrates. J. Adhes Dent..

[86] Queiroz J.R., Massi M., Nogueira L., Sobrinho A.S., Bottino M.A., Ozcan M. (2013). Silica-based nano-coating on zirconia surfaces using reactive magnetron sputtering: Effect on chemical adhesion of resin cements. J. Adhes Dent..

[87] De Sa Barbosa W.F., Aguiar T.R., Francescantonio M.D., Cavalcanti A.N., de Oliveira M.T., Giannini M. (2013). Effect of water storage on bond strength of self-adhesive resin cements to zirconium oxide ceramic. J. Adhes Dent..

[88] Lung C.Y., Kukk E., Matinlinna J.P. (2013). The effect of silica-coating by sol-gel process on resin-zirconia bonding. Dent. Mater. J..

[89] Subasi M.G., Inan O. (2014). Influence of surface treatments and resin cement selection on bonding to zirconia. Lasers Med. Sci..

[90] Kern M., Passia N., Sasse M., Yazigi C. (2017). Ten-year outcome of zirconia ceramic cantilever resin-bonded fixed dental prostheses and the influence of the reasons for missing incisors. J. Dent..

[91] Rathmann F., Bomicke W., Rammelsberg P., Ohlmann B. (2017). Veneered zirconia inlay-retained fixed dental prostheses: 10-Year results from a prospective clinical study. J. Dent..

[92] Chaar M.S., Kern M. (2015). Five-year clinical outcome of posterior zirconia ceramic inlay-retained FDPs with a modified design. J. Dent..

[93] Sasse M., Kern M. (2014). Survival of anterior cantilevered all-ceramic resin-bonded fixed dental prostheses made from zirconia ceramic. J. Dent..

[94] Sasse M., Kern M. (2013). CAD/CAM single retainer zirconia-ceramic resin-bonded fixed dental prostheses: Clinical outcome after 5 years. Int J. Comput. Dent..

[95] Blatz M.B., Vonderheide M., Conejo J. (2018). The Effect of Resin Bonding on Long-Term Success of High-Strength Ceramics. J. Dent. Res..

[96] Luthra R., Kaur P. (2016). An insight into current concepts and techniques in resin bonding to high strength ceramics. Aust. Dent. J..

[97] Inokoshi M., De Munck J., Minakuchi S., Van Meerbeek B. (2014). Meta-analysis of bonding effectiveness to zirconia ceramics. J. Dent. Res..

[98] Otani A., Amaral M., May L.G., Cesar P.F., Valandro L.F. (2015). A critical evaluation of bond strength tests for the assessment of bonding to Y-TZP. Dent. Mater..

[99] Yun J.Y., Ha S.R., Lee J.B., Kim S.H. (2010). Effect of sandblasting and various metal primers on the shear bond strength of resin cement to Y-TZP ceramic. Dent. Mater..

[100] Zhang Y., Lawn B.R., Rekow E.D., Thompson V.P. (2004). Effect of sandblasting on the long-term performance of dental ceramics. J. Biomed. Mater. Res. B Appl. Biomater..

[101] Souza R.O., Valandro L.F., Melo R.M., Machado J.P., Bottino M.A., Ozcan M. (2013). Air-particle abrasion on zirconia ceramic using different protocols: Effects on biaxial flexural strength after cyclic loading, phase transformation and surface topography. J. Mech. Behav. Biomed. Mater..

[102] Özcan M. (2013). Air Abrasion of Zirconia Resin-bonded Fixed Dental Prostheses Prior to Adhesive Cementation: Why and How?. J. Adhes Dent..

[103] Thammajaruk P., Inokoshi M., Chong S., Guazzato M. (2018). Bonding of composite cements to zirconia: A systematic review and meta-analysis of in vitro studies. J. Mech. Behav. Biomed. Mater..

